# Supplementary material for: OtoMatch: Content-based eardrum image retrieval using deep learning
Source: PLoS One. 2020 May 15;15(5):e0232776. doi: 10.1371/journal.pone.0232776 (PMC7228122; doi:10.1371/journal.pone.0232776)
Supplement: Attachment — Submitted filename: Response to Reviewers.docx [file pone.0232776.s001.docx]

**Response to Reviewers**

Reviewer's Responses to Questions

We thank the editor and the reviewers for their thoughtful comments and constructive criticism. We have thoroughly responded to all the comments, and their questions have brought further clarity to the revised manuscript.

5. Review Comments to the Author

Reviewer #1: In this paper, the author proposed a CBIR system for eardrum images which uses deep learning-based approaches to extract the features and creates a lookup table to compare the similarity of query images with training images in multi-dimensional space. The results of the new system are better than other methods.
However, there are some details that should be paid attention.
1. In table 2, the differences between validation accuracy and test accuracy are obvious and sometimes validation is higher and in the other case text accuracy is higher. It is better to explain.

**Answer:**

Table 2. Transfer learning validation and test accuracy values for each fold.

| Fold | Validation Accuracy | Test Accuracy |
| --- | --- | --- |
| 1 | 91.26% | 97.62% |
| 2 | 86.54% | 92.50% |
| 3 | 86.05% | 90.48% |
| 4 | 89.57% | 84.21% |
| 5 | 81.97% | 78.26% |
| 6 | 88.98% | 85.37% |
| 7 | 93.33% | 91.43% |
| 8 | 89.09% | 82.22% |
| 9 | 87.96% | 93.33% |
| 10 | 85.85% | 86.05% |
| Average | **88.06%** | **88.15%** |
| Standard Deviation | **2.87%** | **5.33%** |

The number of images in the test set are three times less than the number of images in the validation set. As a consequence, even when one of the images in the test set is misclassified, the accuracy of the test fold decreases by 2.5%; however, it only decreases by 0.8% in the validation set. For this reason, the results in the validation set are more consistent across folds than those in the test set. This can also be observed in the reported standard deviation values in Table 2. However, the average classification accuracies are comparable 88.06% (±2.87%) for the validation and 88.15% (±5.33%) for the test set. Please see page 14 line 277 for details.

2. The quality of some figures are very poor, especially figure 1, 4 and 5.

**Answer:** As per reviewer suggestion, we have redrawn our previous Figure 1, 4, and 5, which were saved in lower quality by mistake. Please find them in uploaded figures.

3. when comparing the results with traditional methods, all the results are in separate tables. It would be better if you add another table or figure to make it calear.

**Answer:**

This is a really good suggestion. We have combined the results from all methods in Table 6 to reflect this change**.** Please see page 21 line 350 for details.

**Table 6.** Comparison of image retrieval methods. Average accuracies and standard deviations of 1, 3, 5, 7, and 9 content based image retrievals for 10-fold with OtoMatch, traditional KNN, and SVM classifications using handcrafted features.

| Retrieve # Image | Similarity Measurement | OtoMatch | | KNN classification and handcrafted features | | SVM classification and handcrafted features | |
| --- | --- | --- | --- | --- | --- | --- | --- |
|  |  | Average | Standard Deviation | Average | Standard Deviation | Average | Standard Deviation |
| Retrieve1 | Chebychev | 81.14% | 3.94% | 63.68% | 3.68% | 59.92% | 5.19% |
|  | Cosine | 81.71% | 6.87% | 65.59% | 6.81% | 61.00% | 5.40% |
| Retrieve3 | Chebychev | 81.56% | 3.93% | 64.22% | 4.35% | 60.36% | 2.25% |
|  | Cosine | 82.33% | 4.08% | 62.78% | 4.63% | 59.92% | 3.34% |
| Retrieve5 | Chebychev | 81.18% | 4.05% | 61.95% | 4.11% | 58.29% | 2.87% |
|  | Cosine | **82.60%** | **3.89%** | 61.18% | 3.87% | 57.06% | 2.02% |
| Retrieve7 | Chebychev | 81.79% | 4.13% | 60.07% | 4.31% | 57.23% | 3.45% |
|  | Cosine | 82.47% | 4.12% | 59.43% | 3.81% | 56.41% | 3.53% |
| Retrieve9 | Chebychev | 81.36% | 4.42% | 58.61% | 3.72% | 55.42% | 3.54% |
|  | Cosine | 82.26% | 3.66% | 59.19% | 3.50% | 56.27% | 2.83% |

Table 6 combines the averages and standard deviations of 10-fold results of CBIR systems OtoMatch, KNN and SVM classification with handcrafted features for 1, 3, 5, 7, and 9 retrieve images with Chebychev and Cosine similarity measurements.
